# Supplementary material for: Patterns of Intron Gain and Loss in Fungi
Source: PLoS Biol. 2004 Nov 30;2(12):e422. doi: 10.1371/journal.pbio.0020422 (PMC532390; doi:10.1371/journal.pbio.0020422)
Supplement: Table S1 — Also available at http://genes.mit.edu/NielsenEtAl/. (4.3 MB ZIP). [file pbio.0020422.st001.zip › NielsenEtAl/html/363.html]

AN2299.1.NCU07739.1.MG05336.1.FG02008.1


```
 CLUSTAL W (1.82) Multiple Sequence Alignments - Introns Inserted


Sequence 1: NCU07739.1	520 aa
Sequence 2: FG02008.1	469 aa
Sequence 3: AN2299.1	509 aa
Sequence 4: MG05336.1	509 aa
Alignment Length: 549 aa
Number Identitical Residues: 194 aa
Alignment Score (without introns) 10654


MG05336.1 	MAWSRIGMKQRGPMPVASVSSLLPRRLLPSSASSVFTNGAWYLIAAVLLFAPMGSGHEHH
NCU07739.1	MTLGNHPADGGGGGGCGSSSSSLS---FKVPRVPSYITTTAATAVLAAAFIPMASGHDHH
FG02008.1 	--------------------MVVT---YKT---KAFAAPSLFSIAIIALLAPVTQAHEHD
AN2299.1  	-----------------------------------MLGALRLRGVLGALLLASPALAHGD
          	                                            .    : .     . .

MG05336.1 	QEKIQEGEAISMDPI0-----------MLAFGVLFPLGMVLG0LVKSRWHVPVQTIASAI
NCU07739.1	EDQIPEGATISVEPL~DTTLWLHIFLQTFVFGILFPTGMVLG0IVKSRWHVPLQVLATAL
FG02008.1 	TSKIPEGESVSLEPL0DTILWIHILIQMLAYGVIFPVGMVLG0MTKNRWHVPTQVVGTIL
AN2299.1  	HSHVPEGAAISDDPI0DSTLWIHMILMGVSFGIIFPLGMVLG0IIRSRWHVPVQVIGTAI
          	 .:: ** ::* :*: .:          . :*::** ***** : :.***** *.:.: :

MG05336.1 	AILGAILGHSHGGREFAYS-AHAGFADVVYGLLFVQIVLGVYLKLHIERGFLGRIRRWLV
NCU07739.1	ALLGYALGHLHKGRQFVSNNVHASFANILFLMMLAQVVLGVYLKLHLEKGFQGRIRPFFR
FG02008.1 	AILGFFLGHAHKGRQFIPNHVHGKFAHVLQLTLAAQVILGLYLKGHWEKGINGKIRKLIR
AN2299.1  	AVVAYFLGHAHKGRQFAKN-VHASFANWLMFFLAAQVILGVYLKLHIEKGILGKFRKILV
          	*::.  *** * **:*  . .*. **. :   : .*::**:*** * *:*: *::*  : 

MG05336.1 	VAHGINGKLFPVVSWVQLLFGGIAALGFCHDDHLGQCVAHFIMGSAFIAYGTLLTIMLLA
NCU07739.1	ILHSINGKAFPLAAWVQMVFGGITALGFCQGDHLGQCLAHFIMGSAFIAYGVLLTIVLLV
FG02008.1 	PCHSIIGKLMPILAWTQMIFGGITALGFCQGDHTGQCAAHFIMGGAFIAYGILLTILLVV
AN2299.1  	LLHGILGKLMPILSWVQMLFGGITSLGFCRDDHLGQCLAHFIMGSAFIAYGILLTILLLV
          	  *.* ** :*: :*.*::****::****:.** *** ******.****** ****:*:.

MG05336.1 	GQLWLRRTGRSQEFFDSAIIAAWGCVNTFTEHRWGEEWRGNDWQHTAMGIVWWCAGLAGI
NCU07739.1	GQLWLRRTGRSQEFFDSAVIAAWGCVNTFTEHRWGTAWVRNDWQHTTMGVIWWAAGLAGI
FG02008.1 	GQLWIRRNGRSQEFYDSATIAAWGCINTFTEHRWGTDWVKNDWQHTTMGVIWWSAGLVGI
AN2299.1  	GQYWLRRTGRSQEFFDSLVIAAWGCVNTFTEHRWGGPWVHNDLQHTTMGIVWWCAGLLGI
          	** *:**.******:**  ******:*********  *  ** ***:**::**.*** **

MG05336.1 	WLSRGRDGSPKRNFIPGFVLILTGWSMSAHTQHSMASSALHSMFGYTLMAAGVTRIIEVS
NCU07739.1	WLSKNRDGTPKRNFIPGFVLLMTGWAMSAHPQELMISAMTHKMFGYSLMGAGLTRIIEIS
FG02008.1 	WLSRDRDGNPKRNFIPAFVIFITGWGMSAHPQELMVSAATHTMFGYTLMGVGITRVIEIA
AN2299.1  	WLSRKRNGRPKRNLIPAIVILLTGYAMSGHPQHSMISTMIHKIFGYTLMAAGLTRIIEIS
          	***: *:* ****:**.:*:::**:.**.*.*. * *:  *.:***:**..*:**:**::

MG05336.1 	FVLQDKTGVSTAGDEVNSFQYVPVF0LLFASGYLFMGATEEQMALLESSPMDHVAYILIL
NCU07739.1	FILKDKPAVSDQGYETHSFQYVPVF0LLYASGFLFMSATEEQMALVAGSGIDHVAYVLII
FG02008.1 	FVLRDAHSISEDGRSANSFQFIPCF0LMYAAGFLFMGATEEQMMLVASSSMDHISYILIT
AN2299.1  	FVLRDRSSVSADGSDPNSFQYLTPF0LLYASGFLFMGATEEQMMLLENAGITHVSYVLIL
          	*:*:*  .:*  * . :***::. * *::*:*:***.****** *: .: : *::*:** 

MG05336.1 	YSLAFLTFLF~VCMLLHVY---------DRASYN-----SDAYKNLT-------------
NCU07739.1	YSMAFILFLF~VNILIHLY---------DRGANADGLNKNTSAQHGNGAIRLN------G
FG02008.1 	YSLASLTFLF~VNILIGLW---------DRLAHP----VLDTKELGN-------------
AN2299.1  	YSIAFILFLF1VNILLHIYAVHAWPESADPAAHSHSRISTDISDQGPSGSIALPNGPGSS
          	**:* : *** * :*: :::  : ..::*  :   .       .   ..    .....:.

MG05336.1 	-AANTRDDAVIESERRRLQDVEEFELDGLMTDDEDDEEAAKRKLLSKEKDVEGSTSTGPG
NCU07739.1	RATSTNPVAQ---QQQQLRDAGEFELEGLMSDEEDDDEVAARRNLLKN---EGEGLGVAS
FG02008.1 	-NRYANGLTV---EDGQMRDAQEFELDGLTSDDDDD----PSRQMLN-------------
AN2299.1  	RFTHVNGHARTPSEAQRIQDAETFELQGLISDEEEDEDKVPPYEGRRH---GPGPRKAED
          	    ..  :   ::  :::*.  ***:** :*:::*..        .            .

MG05336.1 	SPSTVGRNSDRVA
NCU07739.1	PGTTAARR-----
FG02008.1 	--SSNARN-----
AN2299.1  	EESAPLFRKDRE-
          	  ::   ...
```
